# Pro-Resolving Factor Administration Limits Cancer Progression by Enhancing Immune Response Against Cancer Cells

**DOI:** 10.3389/fimmu.2021.812171

**Published:** 2022-01-18

**Authors:** Audrey Wetzel, Francis Bonnefoy, Cécile Chagué, Mathieu Vetter, Mélanie Couturier, Blandine Baffert, Olivier Adotévi, Philippe Saas, Sylvain Perruche

**Affiliations:** ^1^ University of Bourgogne Franche-Comté, INSERM, EFS BFC, UMR1098, RIGHT Interactions Greffon-Hôte-Tumeur/Ingénierie Cellulaire et Génique, LabEx LipSTIC, Besançon, France; ^2^ MED’INN’Pharma, Besançon, France; ^3^ Department of Medical Oncology, University Hospital of Besançon, Besançon, France

**Keywords:** cancer, inflammation, pro-resolving factors, anti-tumor response, macrophages

## Abstract

Cancers are consequences of cellular dysfunction leading to an aberrant cellular multiplication and proliferation, subsequently yielding metastasis formation. Inflammatory reaction, with immune cell recruitment, is the main defense against precancerous lesions. However, an inflammatory environment also favors cancer cell progression, with cancer cell evasion from immune surveillance, leading to cancer development. Current therapeutic strategies enhance this natural immune response in order to restore immunosurveillance. The variety of these strategies is a predominant source of inflammatory mediators used by cancer cells to grow, differentiate, and migrate, therefore encouraging metastasis formation. For this reason, during cancer progression, limiting inflammation appears to be an innovative strategy to avoid the escape of cancer cells and potentially enhance the efficacy of antitumor therapies. Thus, this study aims to investigate the impact of administering pro-resolving factors (SuperMApo^®^ drug candidate), which are inducers of inflammation resolution, in the framework of cancer treatment. We have observed that administering pro-resolving mediators issued from apoptotic cell efferocytosis by macrophages controlled peritoneal cancer progression by limiting cancer cell dissemination to the blood and mesenteric lymph nodes. This observation has been linked to an increase of macrophage mobilization in both peritoneal cavity and mesenteric lymph nodes. This control is associated to a restricted immunosuppressive myeloid cell circulation and to an IFN-γ-specific anti-tumor T-cell response. Altogether, these results suggest that administering proresolving factors could provide a new additional therapeutic alternative to control cancer progression.

## Introduction

Inflammation is a natural process of the body to fight not only against aggressions such as infections but also against cancer cells. Naturally, inflammation is characterized by an inflammatory response, which self-resolves allowing return to homeostasis (1–5). Resolution starts with the accumulation of neutrophils mainly within the aggressed tissue and, once their job is done, for want of nutriments these cells commit to apoptosis. In addition to a lipid switch from pro-inflammatory lipid production to specialized proresolutive lipid mediators (SPM) synthesis, apoptotic cell-derived factors and the factors released by phagocytes eliminating apoptotic cells initiate the resolution process. This limits innate cell infiltration, enhances efferocytosis, and favors return to homeostasis. Immunosurveillance consists in the recognition by the immune system of tumor-derived antigen that induces a tumor antigen-specific immune cell response that eliminates cancer cells (6–8). The first key cells contributing to this elimination are myeloid cells (9, 10). Resident cells (macrophages and dendritic cells) react to tumor antigens and initiate inflammation by secreting proinflammatory mediators, such as cytokines (IL-1β, IL-6, TNF-α) (11). This inflammatory microenvironment favors other immune cell recruitment, such as monocytes, which differentiate into inflammatory cells. In the tumor, activated myeloid cells capture tumor-derived antigen and migrate to lymphoid organs in order to present the antigen and stimulate T cells, enhancing adaptive immunity against cancer cells, and ultimately kill them at the tumor site (12–15).

Although inflammation is a necessary step for immunosurveillance, persistent and non-resolved inflammation allows cancer to progress. Intrinsic inflammation during immunosurveillance is a source of pro-inflammatory mediators (cytokines, DAMPs, growth factors) (16, 17). This rich inflammatory environment is used by cancer cells to progress by growing, migrating and therefore encouraging metastasis formation (16–18). Finally, this escape favors an immunosuppressive environment not only from cancer cells but also immune cells, leading to a global shutdown of the immune system (named cold tumor).

However, current therapeutic strategies (radio-, chemo-, and immunotherapies) target this cold tumor to restore an immunosurveillance stage (19–21). Unfortunately, these approaches are also responsible for a rich inflammatory environment, which contributes to the persistence of tumor cells (cytokine syndrome release, metastasis, therapy resistance) (16, 17, 22–27). This observation was carried out in 1956, known as the Révész effect (28, 29). Inflammatory response during cancer editing and anti-cancer therapy is still controversial today (30–32). The combination of anti-inflammatory drugs with biologics targeting inflammation has been shown to reduce cancer cell proliferation and angiogenesis (32–37). For instance, a meta-analysis reported in 2017 that nonsteroidal anti-inflammatory drugs reduced the risk of distant metastasis notably in prostate and breast cancer (38). Anti-IL-6 or anti-TNF-α antibodies are respectively associated not only to a decrease of cytokine production, thus avoiding immune cell recruitment and angiogenesis, but also to a decrease of an immunosuppressive protein (PD-L1) favoring T-cell infiltration (11, 39–42). However, these different approaches, which consist of inflammation antagonists, could cause side effects and are not equivalent to inflammation resolution approaches (32, 33, 36, 39). Targeting pro-resolving phenotypic switches rather than using pro-inflammatory inhibiting compounds will likely provide an alternative approach that may hold greater benefit for cancer treatment. Many pro-resolving mediators exist for which data potentially demonstrate anti-cancer properties (36, 43), but, so far, these have not been translated to the clinic due to their short half-life.

The factors issued from apoptotic cell elimination by macrophages have demonstrated pro-resolutive properties (3). The secretome issued from efferocytosis, also called SuperMApo^®^, is composed of all the factors released by phagocytes eliminating apoptotic cells. These pro-resolving factors, including anti-inflammatory cytokines, growth factors, chemokines, enzymes, and lipids (3), are able to terminate inflammation and initiate tissue healing. SuperMApo has been shown to allow the reprogramming of myeloid cells, notably macrophages, by the action of TGF-β and associated factors, promoting antigen-specific Treg activity (3). Administering SuperMApo resolves the ongoing inflammation and, in particular, ongoing experimental collagen-induced arthritis, thioglycolate-induced peritonitis, and dextran-sulfate-sodium-induced xeno-colitis (3). Interestingly, in experimental cancer models, SuperMApo controls cancer progression by limiting peripheral colonization by cancer cells. This mechanism is associated to an increase of anti-tumor macrophages mobilization, leading to an increase of the antigen-specific T-cell anti-tumor IFN-γ response, and finally limiting the circulation of immunosuppressive myeloid cells. Our data demonstrate that, during cancer progression, targeting inflammation with pro-resolving factors represents an innovative strategy to avoid cancer cell escape.

## Materials and Methods

### Cell Lines and Culture Conditions

The tumor cell lines EL4-luc (defined here as nonimmunogenic) and E.G7-OVA were obtained from the American Type Culture Collection (ATCC, Manassas, VA, USA, respectively, TIB-39-LUC2 and CRL). Cells were cultured following supplier recommendation. The tumor cell line EL4-luc (defined here as immunogenic, UMR1098) was cultured in DMEM (Gibco, Waltham, MA, USA) 10% (*v*/*v*) fetal bovine serum (Life Technologies, Carlsbad, CA, USA), 1% (*v*/*v*) penicillin/streptomycin (Eurobio, Les Ulis, France), 10 mM HEPES buffer (Lonza, Basel, Switzerland), 10 mM nonessential amino acids (Biowest, Nuaillé, France), and 1 mM sodium pyruvate (Sigma-Aldrich, St. Louis, MO, USA). Cells were maintained at an optimal concentration of 0.1 to 1.10e6 cells/mL at 37°C in 5% CO_2_ atmosphere. For *in vitro* study, cancer cells (2.10e4 cells/mL) were cultured with SuperMApo (ratio 1/2) for 24 to 48 h. Cell growth was analyzed by bioluminescence quantification 10 min after addition of 0.075 mg/mL of luciferin (Promega, Madison, WI, USA), viability by staining with fixable viability dye (FVD, Invitrogen, Waltham, MA, USA), and proliferation with Ki-67 (clone 16A8, BioLegend, San Diego, CA, USA). DAMPs were quantified after 24 h of culture at 1.10e6 cells/mL.

### Mice

Female Ly5.2 (Charles River), Ly5.1 (Charles River), and RAGγc^−/−^ C57Bl/6 (in house) mice aged 7–24 weeks were housed in filter-top cages with freely available food and sterile water (Plexx, Elst, Netherlands), at the UMR1098 Animal Facility. All experimental studies complied with European legislation and were approved under projects #2019-001-SP-7PR by the Animal Ethics Committee of Besançon (Comité d’Ethique Bisontin en Experimentation Animale #58) and the French Ministry of Higher Education, Research and Innovation, both authorities for the care and use of animals.

### Lymphoma Model and Tumor Growth Evaluation

C57Bl/6 mice received EL4 cells (2.10e6 cells/20-g mouse [immunogenic EL4 cell line] or 2.10e4 cells/20-g mouse [non-immunogenic EL4 cell line]; i.p.) in 1 mL of phosphate-buffered solution (PBS). The number of cancer cells was adapted according to the weight of the mice on the day of injection. Tumor progression was followed by bioluminescence quantification in the 10 min following the i.p. injection of 15 mg/mL of luciferin (Promega) and analyzed by IVIS Lumina III Series & Living Image Software (PerkinElmer, Waltham, MA, USA).

### Treatments

SuperMApo was produced following previously described methods (3). Briefly, mouse thymic cells were irradiated (35 X-Gray, Raycell blood irradiator; Best Theratronics, Ottawa, ON, Canada) to induce cell apoptosis. Irradiated cells were cultured for 6 h in Dulbecco’s modified Eagle’s medium supplemented with 10% heat-inactivated fetal calf serum (FCS; Life Technologies), 1% penicillin/streptomycin, 10 mM HEPES buffer (Sigma Aldrich), and 10 mM nonessential amino acids (Invitrogen). Cell apoptosis was determined by flow cytometry using positive annexin-V staining and negative 7-AAD nucleus staining (BD Bioscience, Franklin Lakes, NJ, USA). Macrophages were isolated from peritoneal cavity lavage with HBSS after a 48-h mobilization by thioglycolate injection (3%/mL/mouse; i.p.). Peritoneal lavage cells were then washed and cultured for 6 h in RPMI supplemented with 10% FCS, 1% penicillin/streptomycin, 10 mM nonessential amino acids (Invitrogen). Apoptotic cells and macrophages were finally cultured together to a 5:1 ratio for 48 h in RPMI supplemented with 2% FCS, 1% penicillin/streptomycin, and 10 mM nonessential amino acids (Invitrogen), and the culture supernatant was collected, centrifuged, 0.22 µm filtrated, and stored at −80°C. The supernatant corresponds to SuperMApo.

SuperMApo or vehicle (RPMI) was administered at day 0, 4, or 7 after EL4 injection. The mice received 1 mL of SuperMApo or vehicle repeated 48 h later, intraperitoneally.

### Cell Sorting, Culture, and Analysis

Cells were extracted from lymphoid organs or blood or peritoneal lavages and stimulated for 4 h with 100 ng/mL of phorbol 12-myristate 13-acetate (PMA, Sigma-Aldrich), 4 µg/mL of ionomycin (Sigma-Aldrich), and 4 µl/mL of Golgi Plug (BD Biosciences) for T-cell intracellular staining, or with 1 µg/mL of LPS (*Escherichia coli* O55:B5, Sigma) and 1 µl/mL of Golgi Plug (BD Biosciences) for antigen presenting cell intracellular staining. Cells were stained for CD4 (clone RM4-5, BD Biosciences), CD8 (clone 53-6.7, BD Biosciences), CD11b (clone M1/70, BD Biosciences), CD45 (clone 30-F11, BD Biosciences), CD45.1 (clone A20, BD Biosciences), CD45.2 (clone 104, BD Biosciences), CD80 (clone 16-10A1, BD Biosciences), CD86 (clone GL-1, BioLegend), CD152 (clone UC10-4F10-11, BD Biosciences), CD206 (clone C068C2, Sony, Tokyo, Japan), F4/80 (clone T45-2342, BD Biosciences), Ly-6G and Ly-6C (clone RB6-8C5, BD Biosciences), I-A/I-E (clone M5/114.15.2, BioLegend), IFN-γ (clone XMG1.2, BD Biosciences), IL-6 (clone MP5-20F3, BD Biosciences), Ki-67 (clone 16A8, BioLegend), Ly-6C (clone REA796, Miltenyi, Bergisch Gladbach, Germany), Ly-6G (clone REA526, Miltenyi), OVA (25-D1.16, Invitrogen), PD-L1 (clone 10F.9G2, BioLegend), and TNF-α (clone MP6-XT22, BD Biosciences) expression. For intracellular staining, cells were permeabilized and fixed with Foxp3 kit/transcription factor buffer set (Invitrogen) or BD cytofix/cytoperm (BD Biosciences). Cells were analyzed by flow cytometry using BD LSR Fortessa X-20 (BD Biosciences), using a minimum of 250000 acquired events excluding doublets. Cells harvested from mice were cultured with 35-Gy-irradiated EL4 cells to a 4:1 ratio, or with 10 µg/mL of OVA 257-264 or 323-339 peptides (InvivoGen, Toulouse, France) for 48 and 72 h. Culture supernatants were used for the quantification of IL-6 (BioLegend), IFN-γ (BioLegend), TGF-β (R&D Systems), ATP (Novus Biologicals, St. Louis, MO, USA), HMGB1 (Novus Biologicals), and HSP70 (R&D systems) by ELISA. ELISpot quantification of IFN-γ (Diaclone, Besançon, France) was performed according to manufacturer recommendations. ELISA plates were analyzed using Delfia EnVision (PerkinElmer) and ELISpot plates by Immunospot CTL system (Immunospot, Cellular Technology Limited, Shaker Heights, OH, USA).

### Statistical Analysis

Data were represented as mean ± SEM or individually. Some data pooled from different experiments are expressed as ratio of 100%, where 100% corresponds to the mean of each control group per experiment. Data distribution was studied by normality test (D’agostino and Pearson). Groups were compared with parametric tests (unpaired *t*-test or ANOVA test including multiple comparisons post-tests). For groups unfollowing normality distribution, non-parametric tests were used (Mann-Whitney *U*, one-way ANOVA, or two-way ANOVA tests including multiple comparisons post-tests). A value of *p* < 0.05 was considered with statistical significance using GraphPad Prism version 9 (GraphPad Software).

## Results

### SuperMApo Treatment Controls Cancer Cell Progression

**Figure 1:**
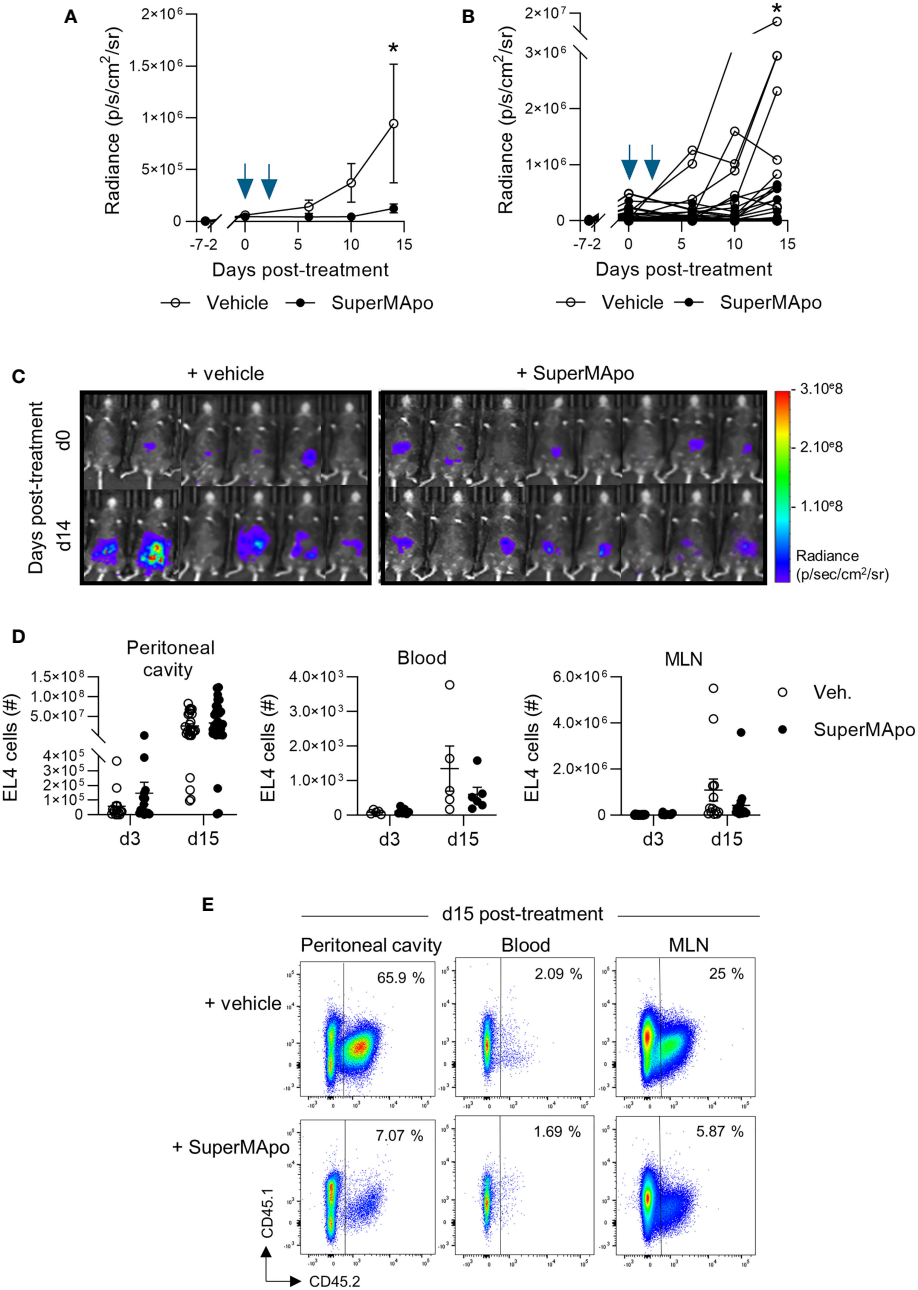
SuperMApo treatment control progression of peritoneal cancer model. **(A)** C57Bl/6 mice received EL4 cancer cells intraperitoneally 7 days before SuperMApo treatment (1 mL, intraperitoneally, twice, at a 48-h interval). Cancer cell progression was followed by bioluminescence evaluation, once to twice a week through luciferin intraperitoneal injection. Radiance is represented as mean ± SEM with 24 to 28 mice per group, or **(B)** individually, and **(C)** bioluminescence representative images at days 3 and 15 post-treatment are also given. Data from 3 independent experiments; ^*^ *p* < 0.05 (two-way ANOVA with Sidak multiple comparisons test). **(D, E)** The number of EL4 cancer cells (CD45.1^−^CD45.2^+^) was determined by flow cytometry at days 3 and 15 post-treatment in the peritoneal cavity, blood, and mesenteric lymph nodes of mice treated with SuperMApo or vehicle (Veh.). Representative examples obtained at day 15 post-treatment are given in **(E)**. Data are shown as individual mouse plus mean ± SEM of 5 to 34 mice per group from 1 to 3 independent experiments; *p* = ns (two-way ANOVA with Sidak multiple comparisons test).

In an experimental model of peritoneal cancer induced in C57Bl/6 mice injected with EL4 cancer cells into the peritoneal cavity, we evaluated the administration of pro-resolving factors (SuperMApo) at the time of cancer detection by luminescence. In this setting, we observed a reduced cancer progression compared with the mice receiving vehicle for the next 2 weeks, as attested by a reduced radiance observed in mice treated with SuperMApo *versus* control (**Figures 1A–C**). Interestingly, the control of tumor growth by proresolving factors was mostly observed in peripheral sites such as blood and mesenteric lymph nodes, where the number of EL4 cells was reduced notably in the mesenteric lymph nodes (**Figures 1D, E** **)**. These data show that SuperMApo treatment limits cancer cell progression by preventing cancer cell progression to secondary sites.

### Pro-Resolving Factors Enhance Anti-Tumor Immune Cell Response

**Figure 2:**
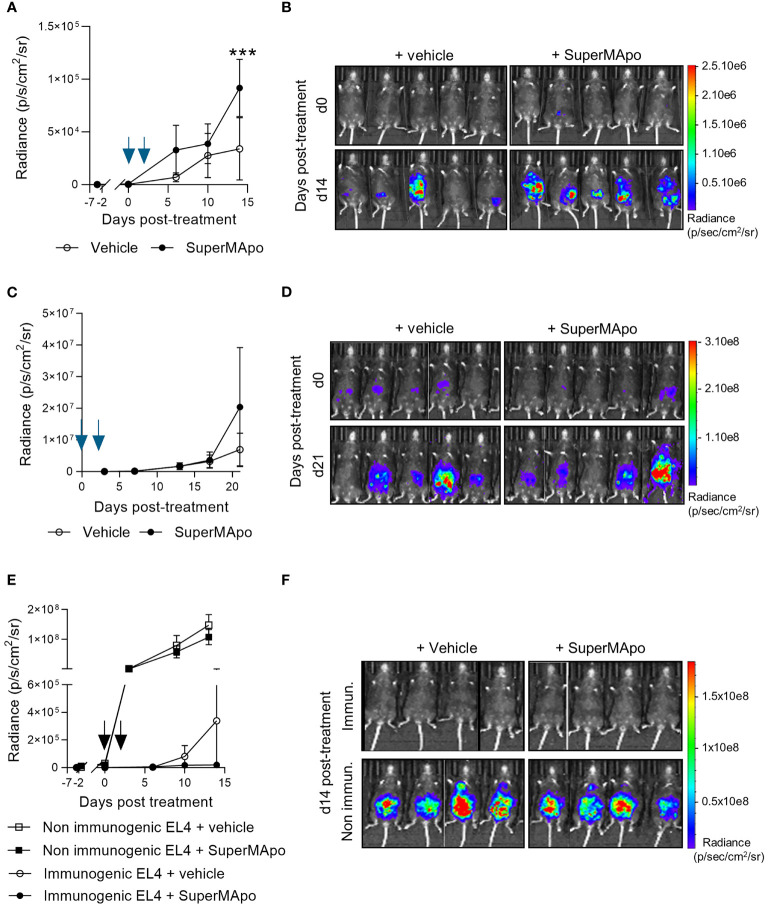
The control of tumor progression is dependent on lymphoid cells and enhances existing antitumor immune response. **(A)** Immunodeficient RAGγc^−/−^ C57Bl/6 mice received EL4 intraperitoneally 7 days before SuperMApo treatment (1 mL, intraperitoneally, twice, at a 48-h interval). Cancer cell progression was followed by bioluminescence evaluation, once to twice a week through luciferin intraperitoneal injection. Data are given as mean of radiance ± SEM with 5 mice per group from 1 experiment, ^***^ *p* < 0.001 (two-way ANOVA with Sidak multiple comparisons test) and **(B)** as bioluminescence images from days 0 and 14 post-treatment. **(C)** EL4-bearing C57Bl/6 mice treated by SuperMApo at day 0 (1 mL, intraperitoneally, twice, at a 48-h interval) the same day as EL4 injection. Data are given as mean of radiance ± SEM with 5 mice per group from 1 experiment, *p* = ns (two-way ANOVA with Sidak multiple comparisons test), and **(D)** as bioluminescence images from days 0 and 21 post-treatment. **(E)** C57Bl/6 mice received EL4 cancer cells intravenously 7 days before SuperMApo treatment (1 mL i.p., repeated after 48 h). Cancer cell progression was followed by bioluminescence evaluation once or twice a week with luciferin intraperitoneal injection. Data are given as mean of radiance ± SEM, 5 mice per group from 1 experiment. *p* = ns (two-way ANOVA plus Sidak multiple comparisons test) and **(F)** as individual mouse bioluminescence images at days 0 and 15 post-treatment.

Going deeper into the mechanisms sustaining the control of tumor growth, we observed *in vitro* that pro-resolving treatment had not a direct effect on EL4 cancer cell growth. Indeed, pro-resolving factors did not affect EL4 cell growth, proliferation and viability *in vitro* (**Supplementary Figure S1** **)**. These data suggest that SuperMApo treatment requires third-party cells to provide anti-tumor activity. Indeed, when the same peritoneal cancer model was used in RAGγc^-/-^ mice (lacking T, B, and NK cells), SuperMApo treatment revealed no anti-tumor effect, as attested by an increased radiance observed in mice treated with SuperMApo *versus* control (**Figures 2A, B** **)**.

Immune cells are necessary to mediate SuperMApo effect. We additionally observed that, to be effective, SuperMApo treatment needs cancer cell-primed immune cells. Indeed, SuperMApo treatment administered to immunocompetent C57Bl/6 mice, before the priming of immune cells by cancer cells (the same day as cancer cell injection), was not able to promote any anti-tumor effect, as attested by an increased radiance observed in mice treated with SuperMApo *versus* control (**Figures 2C, D** **)**. These observations suggest that providing pro-resolving factors during cancer cell priming of T cells enhanced the control of tumor cell growth.

We further addressed cancer cell priming of immune cells by using another EL4 cancer cell line, defined as non-immunogenic. Indeed, this cell line weakly expressed the costimulatory molecules I-A/I-E, CD80, and CD86, the immunosuppressive molecules CTLA-4 and PD-L1, and did not express the DAMPs HSP70, HMGB1, and ATP (**Supplementary Figures S2A–C**). Thus, using this non-immunogenic EL4 cell line, SuperMApo treatment failed to control cancer cell progression while it controlled immunogenic tumor cell line growth (**Figures 2E, F** **)**. Interestingly, in this context, we observed that SuperMApo treatment did not further enhance the immunogenicity of the immunogenic tumor cell line to control its growth (**Supplementary Figures S3A, B**).

Altogether, these data show that the control of intraperitoneal tumor progression by pro-resolving factors is dependent on lymphoid cells primed by immunogenic tumor cells.

### SuperMApo Triggers Early Macrophage Activation

**Figure 3:**
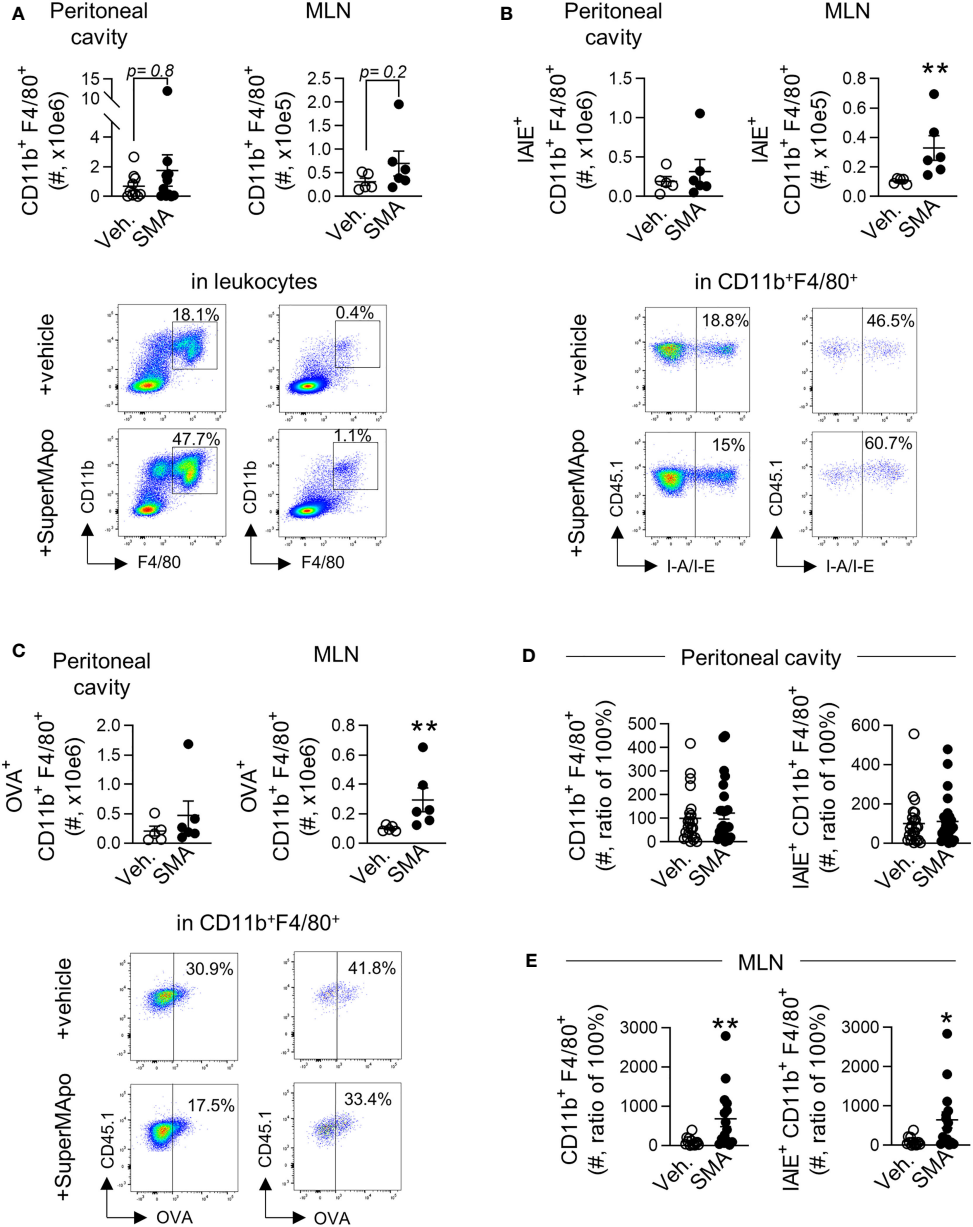
SuperMApo treatment triggers early and sustained macrophage activation. **(A)** Number of macrophages (CD11b^+^F4/80^+^) from C57BL/6 mice bearing immunogenic EL4 cells, treated by SuperMApo (1 mL, intraperitoneal, twice, at a 48-h interval) were evaluated by flow cytometry at day 3 post-treatment in the peritoneal cavity and mesenteric lymph nodes (MLN). Data are given individually plus mean ± SEM of 5 to 11 mice from 1 to 2 experiments; *p* = ns (*t*-test). Representative dot plots are also given. **(B)** The number of mature macrophages (IAIE^+^CD11b^+^F4/80^+^) was also evaluated in the same conditions and are represented as mean ± SEM of 5 to 6 mice per group from 1 experiment; ^**^p < 0.01 (*t*-test). **(C)** Number of macrophages positive for model antigen OVA expression (OVA^+^CD11b^+^F4/80^+^), expressed as mean ± SEM of 5 to 6 mice per group from 1 experiment; ^**^p < 0.01 (*t*-test). **(D)** Number of macrophage subsets CD11b^+^F4/80^+^ and IAIE^+^CD11b^+^F4/80^+^ at day 15 post-treatment in the peritoneal cavity, represented as mean ± SEM of 13 to 14 mice per group from 2 experiments; *p* = ns (*t*-test), and **(E)** in mesenteric lymph nodes, depicted as mean ± SEM of 8 to 9 mice per group from 1 experiment, ^**^ *p* < 0.01 (*t*-test).

Macrophages play an important role in tumor progression and also in the resolution of inflammation (44). We then looked at that population in our model of cancer cell peritoneal injection since they might be preferentially targeted by pro-resolutive factors (3). Three days after the administration of pro-resolving factors, we observed similar numbers of CD11b^+^F4/80^+^ macrophages mobilized in the peritoneal cavity and in the draining lymph nodes in SuperMApo-treated animals and controls (**Figure 3A**). However, we observed a significant predominant number of mature IA/IE^+^CD11b^+^F4/80^+^ macrophages within the draining lymph nodes of tumor-bearing mice treated with SuperMApo (**Figure 3B**). Furthermore, the treatment induced a significant increase, in the draining lymph nodes, of the number of macrophages positive for model antigen OVA harbored by tumor cells (**Figure 3C**). This increased number of mature IA/IE^+^CD11b^+^F4/80^+^ macrophages in the draining lymph nodes was maintained until time of sacrifice, 15 days after treatment (**Figures 3D, E** **)**. Of note, mature macrophages were never observed to be increased in the model with the non-immunogenic cell line despite SuperMApo treatment (**Supplementary Figure S4**). Thus, SuperMApo treatment favors macrophage maturation and presentation of tumor cell-derived antigens.

**Figure 4:**
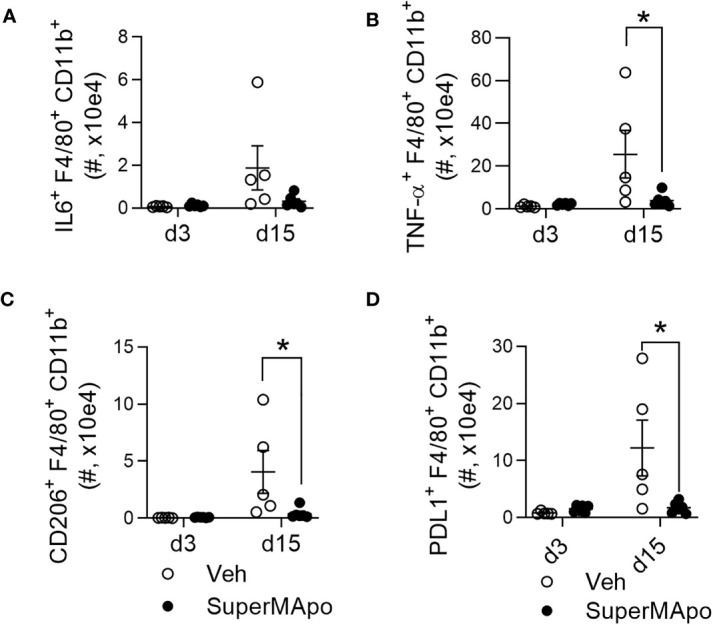
Modifying macrophage subset prevents the emergence of circulating inflammatory and pro-tumor macrophages. **(A)** Number of macrophages (CD11b^+^F4/80^+^) secreting IL-6, from C57BL/6 mice bearing immunogenic EL4 cells and treated by SuperMApo (1 mL, intraperitoneally, twice, at a 48-h interval), were evaluated by flow cytometry at days 3 and 15 post-treatment in blood samples. **(B)** Number of macrophages secreting TNF-α^+^, expressing CD206^+^ **(C)** or PD-L1^+^ **(D)** were also evaluated in the same animals. Data were represented as mean ± SEM of 5 to 6 mice per group from 1 experiment, ^*^ *p* < 0, 05, *t*-test.

Interestingly, the accumulation of mature macrophages presenting the tumor cell-derived antigen within the draining lymph nodes of mice controlling tumor cell growth after SuperMApo treatment was correlated with the reduction of peripheral pro-tumoral macrophages, as attested by significant reduced blood counts of IL-6^+^, TNF-α^+^, CD206^+^, and PDL1^+^ macrophage subsets (**Figure 4** and **Supplementary Figure S5**). In addition, IL-6 concentration was also decreased in plasma and ascites liquid (**Supplementary Figure S5**).

Altogether, these data indicate that SuperMApo treatment targets macrophages, favoring macrophage maturation and their presentation of the tumor cell-derived antigen within the draining lymph nodes and preventing circulation of immunosuppressive pro-tumoral macrophages.

### Activated Macrophages Enhanced T-Cell Anti-Tumor Response

**Figure 5:**
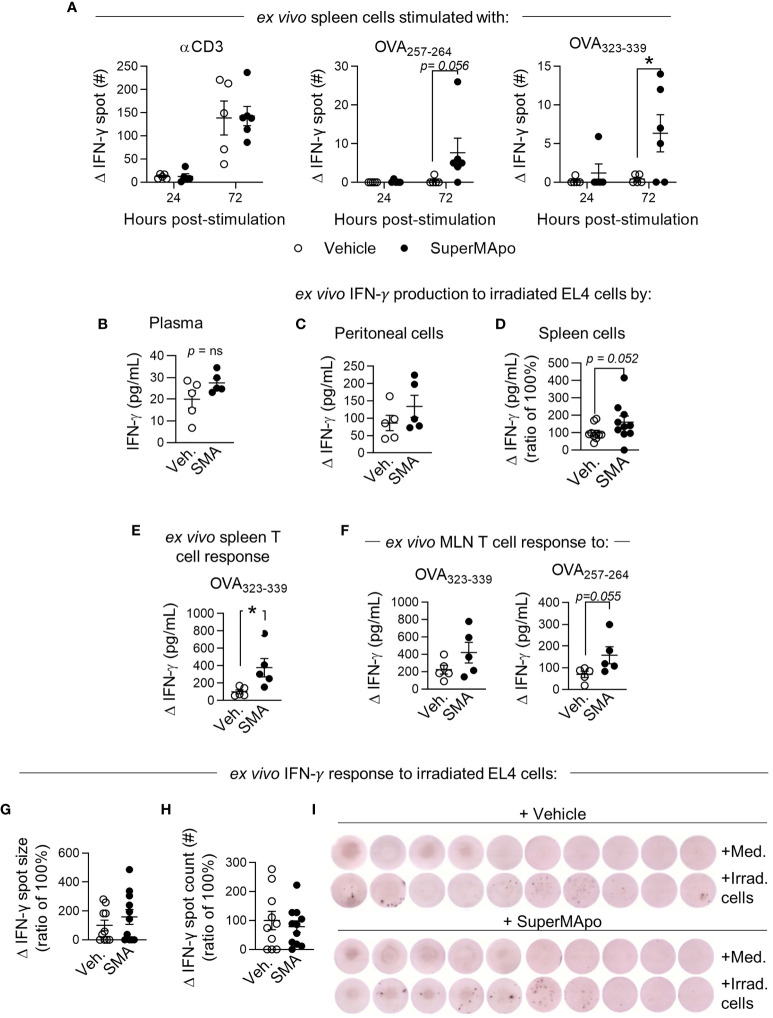
Early type II IFN response is enhanced by SuperMApo treatment and conserved. **(A)** IFN-γ response was evaluated from C57Bl/6 mice bearing immunogenic EL4 cells and treated by SuperMApo (1 mL, intraperitoneally, twice, at a 48-h interval). Three days post-treatment, splenocytes were stimulated for 24 or 72 h with α-CD3 or OVA_257–264_ (for CD8^+^ response) or OVA_323–339_ (for CD4+ response). IFN-γ spot delta between non-stimulated (medium) and stimulated cell response is evaluated by ELISpot, mean ± SEM of 5 to 6 mice per group from 1 experiment, ^*^ *p* < 0.05, two-way ANOVA + Sidak multiple comparisons test. **(B)** At day 15 post-treatment, IFN-γ was quantified by ELISA in plasma, mean ± SEM is represented by 5 mice per group from 1 experiment, *p* = ns, *t*-test; **(C)** in the peritoneal cavity, cells are stimulated for 72 h with irradiated EL4 (4:1), delta of response *versus* unstimulated cells (medium), mean ± SEM of 5 mice per group from 1 experiment, *p* = ns, *t*-test; **(D)** in spleen, cells are stimulated for 48 h with irradiated EL4 (4:1), delta of response *versus* unstimulated cells (medium), mean ± SEM of 10 mice per group from 2 experiments, *p* = 0.052, *t*-test. **(E)** Quantification of T-cell response was evaluated in spleen with OVA_323–339_ stimulation for 72 h, delta of response *versus* unstimulated cells (medium), mean ± SEM of 5 mice per group from 1 experiment, *p* = ns, *t*-test, **(F)** in mesenteric lymph nodes (MLN) OVA_323–339_ or OVA_257–264_, delta of response *versus* unstimulated cells (medium), mean ± SEM of 5 mice per group from 1 experiment, *p* = ns, *t*-test. **(G, I)** IFN-γ response was evaluated by ELISpot with spleen cells of mice bearing immunogenic EL4 cells and treated by SuperMApo (1 mL, intraperitoneally, twice, at a 48-h interval) 15 days post-treatment, stimulated with irradiated EL4 cells (4:1 ratio), for 72 h. Data are shown as the delta of IFN-γ spot size and count of stimulated minus unstimulated cells (medium) and expressed as mean ± SEM of 9 to 10 mice per group from 2 independent experiments. *p* = ns (*t*-test). **(I)** Representative images of IFN-γ spots from G.

Because macrophages demonstrated enhanced presentation of the tumor cell-derived antigen, we then focused our study on T cells. Three days after treatment, at the time when we observed mature tumor antigen-presenting macrophages, we observed an enhanced T-cell response in the spleen. Indeed, we observed an OVA-specific CD4^+^ and CD8^+^ IFN-γ T-cell response only in mice receiving SuperMApo (**Figure 5A** and **Supplementary Figure S6**). This SuperMApo-induced tumor-specific T-cell response was maintained over time, as 15 days post-treatment, IFN-γ was quantified higher in the plasma of treated mice, but not at a significant level (**Figure 5B**). In addition, an IFN-γ response was observable at that time point, 15 days post-treatment (**Figures 5C, D** **)**, which is associated to enrichment of antigen-specific IFN-γ^+^ T cells in the spleen and mesenteric lymph nodes of SuperMApo-treated mice (**Figures 5E, F** **)**. Interestingly, this increase of the IFN-γ response seemed to be associated to a higher capacity of T cells to release IFN-γ but not to an increase in the proportion of IFN-γ^+^ T cells (**Figures 5G–I** and **Supplementary Figure S7**). Altogether, these data show an early and enhanced IFN-γ T-cell response post-SuperMApo treatment, specific to tumor antigens.

## Discussion

Here, we provide data showing that pro-resolving factor administration was able to control cancer progression, in particular, the dissemination of cancer cells by enhancing an existing immune response. We have shown that SuperMApo treatment involved macrophage mobilization and activation. Macrophage deployment was associated with an increase of the IFN-γ^+^ T-cell anti-tumor response. Additionally, we have demonstrated that enhancing an existing immune response with pro-resolving factors prevented pro-tumoral macrophage circulation. This highlights the use of pro-resolving factors, especially here SuperMApo, as a new therapeutic approach against cancer propagation.

First, we demonstrated that pro-resolving factor administration controlled peritoneal cancer progression in the peritoneal cavity but more importantly limiting cancer cell progression outside the peritoneal cavity. Indeed, SuperMApo treatment prevented cancer cell accumulation in the blood and in the mesenteric lymph nodes. To date, only few studies have evaluated the benefits of using pro-resolving factors to target cancer cell progression and have been restricted to the use of specialized pro-resolving lipid mediators (SPM). Resolvins have been shown to suppress tumor growth (36, 43, 45–51). These observations have been mostly done in solid cancer models, but few have been done on liquid cancer (45). Interestingly, resolvins have been shown to be involved in metastasis formation. Mice knocked out for the resolvin receptor ALX/FPR2 demonstrated a higher spontaneous metastasis growth after tumor resection than wild-type mice (45), and resolvin injection after intravenous B16 cell injection, limited metastasis formation (45). While the SPM content of SuperMApo is limited (data not showed), triggering resolution by this complex biological product also limited cancer progression. Indeed, only arachidonic and docosahexaenoic acid have been quantified in SuperMApo, which may initiate the synthesis of SPM *in vivo*, participating in controlling tumor cell escape as described for resolvin. While pro-resolving mediators have failed to be translated into the clinic due to their short half-life, SPM precursors might represent a better therapeutic alternative.

The control of inflammation associated to cancer therapy by pro-resolutive drugs such as SPM has proved successful in enhancing cancer therapy efficacy (44, 49). In our hands, it is important to note that SuperMApo pro-resolutive factors are only able to sustain anti-tumoral immunity in a context of an immunogenic tumor and a primed immune system. This suggests that indeed, inflammation associated either to the tumor or to the treatment, is the condition of success for pro-resolutive approaches to sustain anti-tumoral activity. Thus, SuperMApo needs to be evaluated as a co-therapy to promote the control of tumor growth.

Also, we observed that the anti-tumor effect of SuperMApo was dependent on existing host immune cell response specific to cancer cells. Myeloid cells, and macrophages even more, are both sentinel cells controlling tissue hemostasis as well as regulators of pro-tumor inflammation due to their plasticity (52, 53). Indeed, macrophages have pro- and also anti-tumor properties (18, 52–54). Tumor-associated macrophages (TAM) are associated with a poor prognosis in cancer (18, 25, 29, 52, 53, 55, 56). TAM controlled circulating cancer cell recruitment to the primary tumors and are involved in the metastatic cascade (29, 53, 55, 57, 58). Several studies have shown that targeting macrophages improved cancer outcome, either by depleting them with clodronate liposome injection for instance, blocking the phagocytosis pathway, inhibiting their recruitment (CXCR2/CCL2 pathway blocking), targeting CD47, or by reprogramming them toward an anti-tumoral phenotype (11, 25, 52, 53, 57, 59). Anti-inflammatory approaches in cancer, such as aspirin, have been shown to trigger SPM production that stimulates cancer resolution by targeting macrophage subsets (36). Specialized pro-resolving lipid mediators have been described to favor the switch of TAM with a pro-inflammatory phenotype to pro-resolving macrophages limiting CD206 expression, TNF-α, and IL-6 secretion (36, 48, 52, 60). In addition, pro-resolving mediators such as SPM can trigger an increase of phagocytosis from macrophages (3, 36, 45, 52). Macrophages are thus key cells in cancer progression, from escape to immune surveillance. We have demonstrated that SuperMApo pro-resolving properties allowed the reprogramming of antigen-presenting cells (APC) (3). Here, we show that in a peritoneal cancer model, SuperMApo treatment triggered early mobilization and activation of macrophages. Interestingly, this conversion toward anti-tumor macrophages seemed, over time, to prevent inflammatory circulating and immunosuppressive macrophages. These observations suggest that SuperMApo treatment could enhance an immune surveillance by macrophages without inducing an immunosuppressive environment and, finally, preventing immunosuppressive escape by reducing IL-6 as described (53). Of note, in the context of experimental arthritis, SuperMApo treatment resolved ongoing inflammation through the lasting reprogramming of macrophages with immunomodulatory functions, favoring regulatory T-cell rather Th1 polarization (3). These observations in the context of experimental arthritis and in cancer suggest that together with the modified microenvironment, SuperMApo treatment provides to the macrophages the necessary factors to either escape from inflammation pressure and provides an adapted regulatory response or, acquires the capacity to mount a tumor-derived antigen-specific immune response, respectively. How macrophages adapt their function under SuperMApo treatment remains an ongoing question. Recombinant TGF-β polarizes plasmacytoid dendritic cell (pDC) to favor Th17-cell commitment (61), while factors from efferocytosis, rich in TGF-β, polarize pDC to favor regulatory T-cell commitment (62). Thus, additional researches are needed to better understand what factors and how they could overcome environment-primed macrophage functions.

We also observed that the SuperMApo product was ineffective to control cancer cell progression in the absence of B, T, and NK cells in a RAGγc^-/-^ environment. Indeed, SuperMApo triggered an early IFN-γ T-cell response, sustained over time. Strategies targeting inflammation to prevent an adequate microenvironment for tumor growth and escape have been mostly concentrated on myeloid cells. Again, the depletion of macrophages increased CD8^+^ T cells, and impairment of phagocytosis was associated with an increased IFN-γ T-cell response (57, 59). In murine models, anti-inflammatory drugs have been associated with a more important CD4/CD8 T-cell infiltration in spleen after tumor rejection (49). In contrast, radio-chemotherapy approaches were associated to an increase in the IFN-γ response (19, 26, 63), IFN-γ that is responsible for a pro-tumor TAM phenotype commitment (58). In the context of SuperMApo, composed of pro-resolving mediators including TGF-β and IL-10 (3), we did not inhibit the specific anti-tumor response from T cells. Indeed, SuperMApo injection restored and enhanced a specific anti-tumor IFN-γ response which was correlated to the induction of macrophages highly expressing class II and a tumor-derived antigen. In addition, pro-tumor macrophage phenotype decreased with a low level of IL-6, suggesting the anti-tumor role of the SuperMApo treatment.

In summary, this work demonstrated that SuperMApo disrupted cancer cell progression, preventing cancer cell delocation from the primary tumor site by enhancing a pre-existing anti-tumor immune cell response. SuperMApo treatment triggered macrophage mobilization and activation that enhanced a specific IFN-γ anti-tumor T-cell response, without increasing immunosuppressive myeloid cells. These observations revealed the beneficial impact of pro-resolving mediators in cancer progression. Interestingly, these results opened new opportunities toward a pro-resolving mediator role in cancer treatment. Despite the facts that cancers vary considerably between rodents and humans and that further experimental studies with human material need to be performed, SuperMApo treatment appears promising as a potential new therapeutic strategy to fight cancer.

## Data Availability Statement

The original contributions presented in the study are included in the article/**Supplementary Material**. Further inquiries can be directed to the corresponding author.

## Ethics Statement

The animal study was reviewed and approved by Comité d’Ethique Bisontin en Experimentation Animale #58 and the French Ministry of Higher Education, Research and Innovation authorities.

## Author Contributions

SP and AW conceived and designed the study. AW performed and analyzed the experiments. FB and CC participated in the experiments and helped in the analysis. MV and BB participated in some experiments. MC and PS helped in the design, analysis, and interpretation of some experiments. AW and SP wrote the manuscript. SP and OA supervised the study. All authors discussed the results and the manuscript. All authors contributed to the article and approved the submitted version.

## Funding

This work is supported by LipSTIC LabEx (ANR-11-LABX-0021), MiMedI (grant No. FC0013440), Région Bourgogne–Franche-Comté. AW was supported by a doctoral fellowship from the Association Nationale de la Recherche et de la Technologie (ANRT 2017/1322/2017) with MED’INN’Pharma. CC was supported by a doctoral fellowship from the Ministère de l’Education Nationale, de l’Enseignement Supérieur et de la Recherche.

## Conflict of Interest

FB, MC, PS and SP are shareholders of MED’INN’Pharma SAS.

The remaining authors declare that the research was conducted in the absence of any commercial or financial relationships that could be construed as a potential conflict of interest.

## Publisher’s Note

All claims expressed in this article are solely those of the authors and do not necessarily represent those of their affiliated organizations, or those of the publisher, the editors and the reviewers. Any product that may be evaluated in this article, or claim that may be made by its manufacturer, is not guaranteed or endorsed by the publisher.
