# Supplementary material for: Pro-Resolving Factor Administration Limits Cancer Progression by Enhancing Immune Response Against Cancer Cells
Source: Front Immunol. 2022 Jan 18;12:812171. doi: 10.3389/fimmu.2021.812171 (PMC8804172; doi:10.3389/fimmu.2021.812171)
Supplement: Supplementary file 1 [file DataSheet_1.docx]

Supplementary Material

**B**

Pro-resolving factor administration limits cancer progression by enhancing immune response against cancer cells

Audrey Wetzel^1, 2^, Francis Bonnefoy^1, 2^, Cécile Chagué^1^, Mathieu Vetter^1^, Mélanie Couturier^2^, Blandine Baffert^1^, Olivier Adotevi^1, 3^, Philippe Saas^1^, Sylvain Perruche^1, 2*^

**Supplementary Figure 1. SuperMApo treatment did not act directly on cancer cells. (A)** EL4 cancer cell line (2.10e4 cells/well) was cultured with SuperMApo (or control medium) and bioluminescence was quantified by the radiance after luciferin addition into the culture plate and **(B)** by cell counting. Data are shown as mean ± SEM of triplicates from 3 independent experiments; *p* = ns (One-way Anova test plus Dunn’s post-test). **(C, D)** EL4 proliferation was determined by flow cytometry thanks to Ki-67 staining. Data are represented as mean ± SEM from 3 independent experiments; *p* = ns (t test). **(E)** EL4 cell viability was determined by cell counting in the presence of trypan blue and **(F)** by flow cytometry at 6h and 48h of culture with Fixable Dye Viability labelling . Data are expressed as mean ± SEM from 3 independent experiments: *= *p* < 0,05 (One-way Anova test plus Dunn’s post-test).

**Supplementary Figure 2. Non-immunogenic EL4 tumor cell line was not sensitive to pro-resolving factors-induced anti-tumor immune response.** The mean fluorescence intensity of I-A/I-E^+^, CD80^+^, CD86^+^, CTLA-4^+^ and PD-L1^+^ of was determined by flow cytometry in immunogenic and non-immunogenic EL4 cells, *in vitro* **(A)** and *ex vivo* **(B)** 22 days after injection in C57Bl/6 mice. Data are given as mean ± SEM or with representative dot-plots, from 3 independent experiments; *= *p* < 0,05; ***= *p* < 0,01 (t test). **(C)** DAMPs (HSP70, HMGB1 and ATP) were quantified by ELISA in peritoneal lavage of mice receiving EL4 cell injection 22 days earlier. Data are given in nmol for 1.10e6 cells as mean ± SEM from 1 experiment; *= *p* < 0,05 (t test).

**Supplementary Figure 3. EL4 immunogenicity was not affected by SuperMApo treatment *in vivo*. (A)** The mean fluorescence intensity of I-A/I-E^+^, CD80^+^, CTLA-4^+^ and PD-L1^+^ was determine by flow cytometry *ex vivo* on immunogenic EL4 cells injected in C57Bl/6, 7 days before receiving SuperMApo treatment (1 mL repeated after 48 hrs, intraperitoneal). Data are given as mean ± SEM or representative dot-plots, with 10 mice per group from 2 independent experiments. **(B)** DAMPs (HSP70 and HMGB1) were quantified by ELISA in the peritoneal lavage of the same mice, 15 days after treatment. Data are given as mean + SEM with 5 to 10 mice per group from 1 to 2 independent experiments.

**Supplementary Figure 4. Macrophage subsets were not activated in non-immunogenic EL4 bearing mice after SuperMApo treatment.** CD11b^+^F4/80^+^ and CD11b^+^F4/80^+^IAIE^+^ macrophages subsets were evaluated by flow cytometry at day 15 days post-treatment from the peritoneal cavity (**A**) and in mesenteric lymph nodes **(B)** of C57Bl/6 mice bearing non-immunogenic EL4 cell tumors, treated by SuperMApo (1 mL repeat after 48 hrs, i.p.). Data are given as mean ± SEM with 7 to 12 mice per group from 1 to 2 independent experiments.

**Supplementary Figure 5. IL-6 levels decreased after SuperMApo treatment.** IL-6 quantification was evaluated by ELISA in the peritoneal lavage of C57Bl/6 mice bearing EL4 cell tumors, 3 and 15 days after SuperMApo treatment (**A**; 1 mL, repeated 48 hrs later; i.p.) and in the plasma at day 15 (**B**). Data are shown as mean ± SEM with 5 to 15 mice per group from 1 to 3 independent experiments; *= *p* < 0.05 (t test); **= *p* < 0.01 (Two-way Anova test plus Sidak’s multiple comparisons post-test).

**Supplementary Figure 6. Early IFN response was enhanced by SuperMApo treatment.** IFN-γ response was evaluated from C57Bl/6 mice bearing immunogenic EL4 cell tumors and treated by SuperMApo (1 mL reapted after 48 hrs, i.p.) in the spleen cells, 3 days post-treatment after α-CD3, OVA_257-264_ or OVA_323-339_ stimulation for 72 hrs by ELISpot. Data are given as mean ± SEM with 5 to 6 mice per group or as pictures of wells from 1 experiment; *p* = ns (Two-way Anova plus Sidak’s multiple comparisons test).

**Supplementary Figure 7. Total CD4, CD8, Th1 and Tc1 cells were not modified by SuperMApo treatment.** CD4^+^, CD8^+^ T cells and IFN-γ^+^CD4^+^ and IFN-γ^+^CD8^+^ T cells were analyzed from the spleen (**A**), the peritoneal lavage (**B**) and the mesenteric lymph nodes (**C**) of C57Bl/6 mice bearing immunogenic EL4 cell tumors by flow cytometry 15 days post-treatment with SuperMApo or vehicle (1 mL i.p. repeated 48 hrs later). Data are shown as mean ± SEM with 8 to 34 mice per group from 1 to 7 independent experiments; *p* = ns (t test).
